# Supplementary material for: Nisoldipine Inhibits Influenza A Virus Infection by Interfering with Virus Internalization Process
Source: Viruses. 2022 Dec 8;14(12):2738. doi: 10.3390/v14122738 (PMC9785492; doi:10.3390/v14122738)
Supplement: Supplementary file 1 [file viruses-14-02738-s001.zip › viruses-1963322-supplementary.pdf]

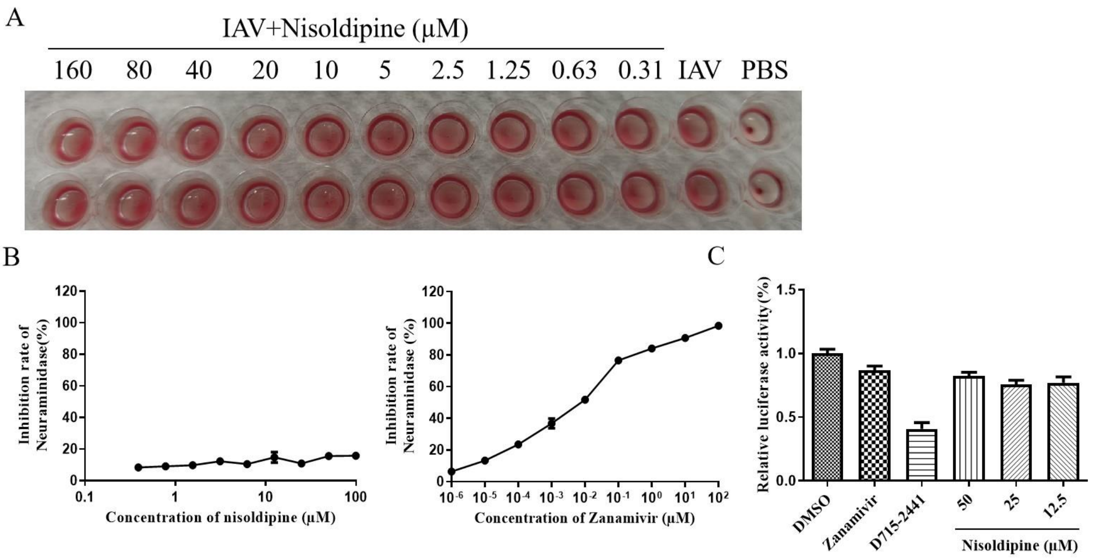

Figure S1. The effect of nisoldipine on other phases during the virus life cycle. (A) To test whether nisoldipine interacted with HA1 by haemagglutination inhibition assay. There is no significant inhibition effect on the agglutination of chicken erythrocytes observed, revealing that nisoldipine was unable to act with the HA1 subunit. (B) Neuraminidase inhibition assay was used to determine whether nisoldipine acted on the release phase of influenza A virus. Zanamivir was a neuraminidase inhibitor as a positive control. (C) The inhibitory effect of nisoldipine on viral polymerase activity was tested by a mini-replicon assay. The results showed that nisoldipine had no significant effect on the polymerase activity. Zanamivir was used as a negative control and D715-2441 as a positive control.
